# A comprehensive quality assessment framework for scientific events

**DOI:** 10.1007/s11192-020-03758-1

**Published:** 2020-11-03

**Authors:** Sahar Vahdati, Said Fathalla, Christoph Lange, Andreas Behrend, Aysegul Say, Zeynep Say, Sören Auer

**Affiliations:** 1 Department of Computer Science, University of Oxford, Oxford, UK; 2 Institute for Applied Informatics (InfAI), Dresden, Germany; 3 University of Bonn, Bonn, Germany; 4 Faculty of Science, University of Alexandria, Alexandria, Egypt; 5 RWTH Aachen University, Aachen, Germany; 6 Fraunhofer FIT, Sankt Augustin, Germany; 7 Institute for Telecommunications (INT), TH Köln, Germany; 8 L3S Research Center, Leibniz University of Hannover, Hannover, Germany; 9 TIB Leibniz Information Centre for Science and Technology, Hannover, Germany

**Keywords:** Recommendation, Scientific events, Quality assessment, Metadata analysis, Bibliometrics

## Abstract

Systematic assessment of scientific events has become increasingly important for research communities. A range of metrics (e.g., citations, h-index) have been developed by different research communities to make such assessments effectual. However, most of the metrics for assessing the quality of less formal publication venues and events have not yet deeply investigated. It is also rather challenging to develop respective metrics because each research community has its own formal and informal rules of communication and quality standards. In this article, we develop a comprehensive framework of assessment metrics for evaluating scientific events and involved stakeholders. The resulting quality metrics are determined with respect to three general categories—events, persons, and bibliometrics. Our assessment methodology is empirically applied to several series of computer science events, such as conferences and workshops, using publicly available data for determining quality metrics. We show that the metrics’ values coincide with the intuitive agreement of the community on its “top conferences”. Our results demonstrate that highly-ranked events share similar profiles, including the provision of outstanding reviews, visiting diverse locations, having reputed people involved, and renowned sponsors.

## Introduction

Scientific publications are the key element of scholarly communication and knowledge exchange and provide a reliable basis for evaluating the academic achievements of researchers (Davidson 2004; Freyne et al. 2010). Current metrics for such evaluations are mainly based on the number of a researcher’s peer-reviewed publications and the number of citations they have received from other peer-reviewed publications (Caires 2015a). Respective quality measures, however, solely represent a very rough approximation of the reality since much relevant information, including details about the review process, the reputation of the reviewers, or the acceptance rate of the chosen publication channel, is simply ignored. This is particularly true for less formal publication venues such as conferences, workshops, or symposia, where a simple citation count is typically not sufficient for determining the quality and impact of an event or event series. The following reasons particularly cause this:

- Non-equal citation rates: Publications of an event, or a journal, have many citations, while others have few, and many have zero (Weale et al. 2004).
- Non-equal citation weights: The overall impact of the citations varies a lot when publications are cited only in the “related work” section of a paper, whereas other publications provide foundations of, e.g., the methodology or motivation section of a paper (Hu et al. 2013; Ciancarini et al. 2013).
- Non-sequential citations: If several publications represent different steps of one overall research effort, the citations of each of them can only be considered individually. There is no way to sum up citations of a sequence of publications of an author (Kumar 2009).
- Non-accepted submissions: There are too many submissions to events or journals and, sometimes, not enough high-quality reviews are carefully made to evaluate each one. The rejected submissions lose possible citations of that particular venue or journal (Terry 2014), i.e., the number of publications cited by this venue goes down.
- Non-discovered publications: Currently, most of the services for discovering related publications use a keyword-based search. All those publications that are not discovered by researchers miss being cited even though the work they report on might be closely related (Remler 2017).

Comprehensive quantification of the quality of scientific events, however, is crucial for researchers because it largely influences the selection of an appropriate event for publishing their results (Bowyer 2012). For lack of better support, researchers traditionally just search for upcoming events that would suit their work-in-progress best in terms of a feasible deadline and a suitable topic; however, quality evaluation largely depends on individual experience or informal knowledge within the community.

There are already attempts to automatically assist researchers in this task by exploring their scientific community or by content-based analysis of the publication lists of related researchers (Luong et al. 2012). The resulting recommendations, however, are often rather superficial and the underlying process neglects the different aspects that are important for authors. For example, authors may prefer certain publishers, indexing services, venue locations, or just want to maximize the likelihood of being published or cited. In addition, the organizers of events receive rather insufficient feedback about the quality of the event (in terms of organization issues, accepted papers quality, etc.), which does not systematically support them in improving the characteristics of their events.

To overcome these deficiencies, we propose to study a broad scope of characteristics of events as a basis for a refined and differentiated quality assessment. To this end, we consider data about the composition of participants, the number of editions, composition of program and organizing committees as well as social aspects, and define corresponding quality metrics. As a result, we present a comprehensive framework of quality assessment metrics, which can be used for ranking, filtering, and recommending events in a flexible and user-defined way. The resulting framework supports the derivation of quality aspects that are relevant for different stakeholders of events, including sponsors, researchers in the roles of authors or participants, publishers, and potential organizers (who want to decide whether to participate in the organizing process or not).

We applied this assessment methodology empirically to 11 high-ranked scientific events in the computer science area (Vahdati 2019)—where conferences are essential (Caires 2015b)—and give suggestions on how the quality estimation process can be systematically improved this way one it has been implemented broadly. We aim at supporting the scientific community in assessing the quality of their events by collecting quality-related metadata in a central database following a standardized structure, on top of which our computable quality metrics can automatically generate rankings and recommendations.

The remainder of this article is organized as follows. Section “Related work” presents related work and provides a systematic overview of systems and tools supporting scholars in generating scientific event-related metadata and analyzing the quality of events. Section “Systematic conceptualization” introduces the fundamental concepts of our systematic definition of event quality. A comprehensive definition of quality dimensions and metrics is presented in section “Quality metrics”. Section “Use cases for quality metrics” explains the possible benefits of such a system for scientific communication. The implementation of the metrics on the OpenResearch.org platform is explained in section “Implementation”. In section “Evaluation”, the result of computing the defined metrics over a representative set of the best CS conferences is presented. Section “Conclusion and future work” concludes and provides general recommendations for different groups of users who could benefit from such a system.

## Related work

**Fig. 1:**
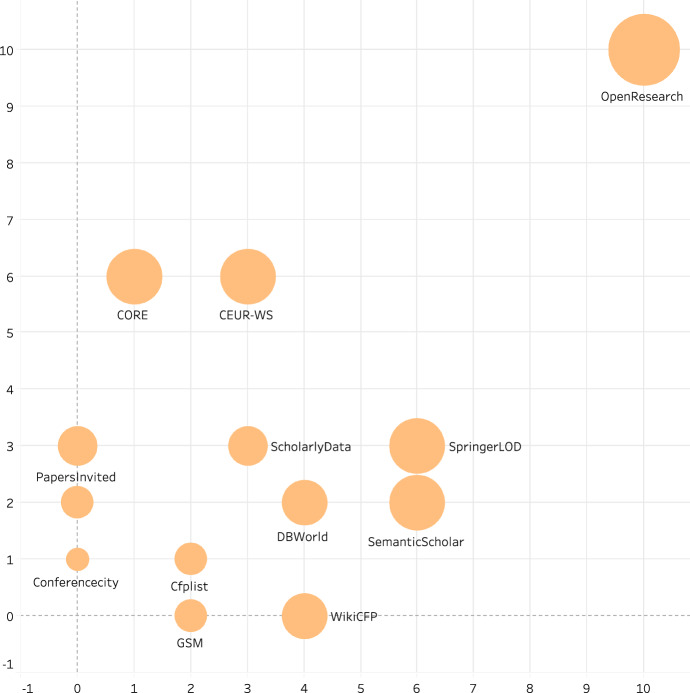
List of services ordered by the extent to which their data is reusable and consideration of quality-related metrics

Bryl et al. mention questions such as “Shall I submit a paper to this conference?”, and point out that the data that is required for answering such questions is not easily available but, e.g., hidden in conference management systems (Bryl et al. 2014). Several efforts of publishing reusable, machine-readable metadata (i.e., *linked open data*^1^) about scientific events have been motivated with quality considerations. As one of the primary purposes of this research work is to propose a comprehensive platform for managing scholarly event metadata, the related services have been evaluated with regard to (1) the re-usability of their data for easy interlinking and (2) the extent to which they consider quality-related metrics. Figure 1 depicts the distribution of such services in these two dimensions.

The Springer LOD^2^ is a dataset about conference proceedings—published by the Springer publishing house, e.g., in the Lecture Notes in Computer Science series—for public reuse. However, the number of event properties considered is limited to basic metrics such as event title, date, location, and this dataset does not adequately cover quality-related properties. Similarly, *ScholarlyData*^3^ provides RDF dumps for scientific events (Nuzzolese et al. 2016). The scientific events ontology (OR-SEO) represents scholarly event metadata in a semantically enriched form by integrating existing events vocabularies and making explicit the relationships and interconnections between event data (Fathalla et al. 2019b). This representation greatly supports the transformation of from a “Web of documents” into a “Web of data” in the scientific domain. In addition to representing the time and place of a scholarly event, OR-SEO also represents the roles of involved agents at particular events. OR-SEO is used represent events metadata in the EVENTSKG (Fathalla and Lange 2018; Fathalla et al. 2019a) dataset, a 5-star dataset containing metadata of 73 top-ranked event series belonging to eight computer science communities took place in the past 50 years. Conference-Ontology, a new data model developed for ScholarlyData, improves over already existing ontologies about scientific events such as the Semantic Web Dog Food (SWDF) ontology. Our own ongoing work on extracting linked data from the CEUR-WS.org^4^ Open-access workshop proceedings volumes are also motivated by quality assessment. In the specific setting of a challenge (SemPub^5^), we ran a few dozens of quality-related queries such as “What workshops have changed their parent conference?” against the linked dataset in order to assess the quality of the workshops published with CEUR-WS.org and to validate different information extraction implementations (Lange and Di Iorio 2014; Di Iorio et al. 2015). Both the work of Bryl et al. and our previous work (Fathalla et al. 2017a, 2018, 2020; Fathalla and Lange 2017) have in common that they lack a systematic, comprehensive definition of quality dimensions and that they focus on just one data source for information about scientific events and therefore only consider such quality metrics that can be computed based on the given data.

The ISO 20121 international standard supports organizers of events of all types—sports, business, culture, politics—in integrating sustainability with their activities.^6^ The standard provides general guidelines and does not explicitly cover scientific events; however, it includes some of the metrics of our model, such as financial support of events through, e.g., sponsoring. The Computing Research and Education Association of Australasia, CORE^7^ uses several metrics in the ranking process such as number of citations, number of submissions and acceptance rates, and the visibility and research track record of the key people hosting and managing the conference as well as the key people hosting the conference. *Google Scholar Metrics (GSM)*^8^ provides another ranking for conferences and journals based on a 5-year impact analysis over the Google Scholar citation data.

Besides, several web resources are devoted to presenting information about scientific events and their quality. *WikiCFP*,^9^ for example, is a repository of calls for papers (CfPs) in science and technology fields. However, WikiCFP does not provide explicit information about the quality of events. Paperleap,^10^ a platform newly established in 2016, tries to support researchers in finding the best opportunities for publishing and presenting their work, however, the service is not free of charge for public use. A similar kind of database but more specifically addressing research in the database field is *DBWorld*,^11^ which is used often as a reference to find the upcoming events of this community. Its data is not easy to be reused (since it is not available in a structured form), and quality metrics are not considered.

OpenResearch.org (Vahdati et al. 201) (more details in section “Implementation”) is a semantic media wiki platform developed based on the quality framework explained in this paper. It considers quality from different aspects and provides an easy way of querying its data. The data is provided both by the community and collected by administrators of the platform. It is made available in CSV and RDF formats for reuse so that it can be replicated or combined in different settings (Wilkinson et al. 2016). OpenResearch.org does not only represent scholarly events but also the scientific contributions within publications (Fathalla et al. 2017b, 2018).

## Systematic conceptualization

A basic terminology of quality, also introducing the essential terms of dimension and metric, is presented in section “Stakeholders and terminology”. The methodology by which we have defined the quality metrics in each dimension is explained in section “Methodology”.

### Stakeholders and terminology

We adopt a broad definition of *quality* as “fitness for use” (Juran 1974; Knight and Burn 2005). Given that scientific events have multiple stakeholders, the quality of an event depends on the perspective of the stakeholder and the context in which an assessment of its quality is required.

- Any potential *participant* may be interested to know the reputation of an event’s keynote speakers and the registration fee.
- *Authors* of submissions, and *publishers* likewise, may be interested in aspects of an event’s peer review process, such as the expertise of the program committee members and the acceptance rate, but also in the long-term impact of publications accepted at the event, as measured by the number of citations they attract over a few years.
- Senior scientists invited to participate in an event’s *organization* may be interested in how long-standing the event’s history is and how many participants it usually has.
- Organizations asked to *sponsor* an event may additionally be interested in the sectors (academia, industry, government, society) the participants come from.

Our further classification of quality indicators follows the standard terminology of *data quality* research, with the key terms of *category*, *dimension* and *metric*. From the informal examples above, it can already be seen that indicators for the quality of an event come from different sources and can thus be classified in different *categories*:

- Some, such as the number of participants, are immediate properties of the *event*.
- Others, such as the reputation of keynote speakers, are properties of *persons* having a role in the event.
- A third category is related to *publications* accepted at the event, e.g., their impact.

Within these broad categories, there exist different quality *dimensions*, e.g., in the “event” category, the quality of its peer-review process, or the quality of its co-located events. The importance of a dimension depends on the context, as pointed out above, for the different stakeholders. The same stakeholder may have changing priorities depending on the situation. For example, the same experienced researcher may not find a conference, with a low acceptance rate, attractive for a paper co-authored with other experienced researchers, whereas the idea of having a paper with a student accepted at the same conference is appealing. Assessing quality with regard to a given metric can have certain advantages or disadvantages, which we discuss in detail for each metric.

Thus, to provide the stakeholders with a versatile toolkit, from which they can flexibly choose what aspects of quality are relevant in their current situation and what weight they should be given in comparison to other aspects, we are aiming at defining a large number of fine-grained quality *metrics* to choose from. Quality metrics are “procedure[s] for measuring a[n ...] quality dimension”, which “rely on quality indicators and calculate an assessment score from these indicators using a scoring function” (Bizer and Cyganiak 2009). Any such metric has a precise definition by which its exact value can be computed from data about the event. If such data is not available, its value can be estimated; if exact computation would take too much time, the value can be approximated. Besides these *objective* metrics, there are also a few *subjective* ones, such as “What reputation does a given person have in a particular community?”.

Further characteristics of a metric include:

- How *easy* is to collect the data, e.g., do we have to calculate the metric from scratch or have others calculated it before, and we just use it, e.g., Twitter hashtags, which are used to raise the awareness for an event through a number of tweets calculated by twitter?
- How easily is the data *available* that would enable the metric’s computation?
- How *reliable* is the data?
- How *precise* is the data?
- How *easy* is the metric to *compute* once the data is known?

### Methodology

In each of the categories introduced above, we established a set of dimensions, guided by the following questions:

- What information is available about events from original sources related to the event? For example, from events’ homepages or calls for submissions.
- What other concepts are related to events? For example, an event takes place in some *location*, and involves *people*.
- In what exact ways are these concepts related to each other? For example, people have different *roles* in an event.

In each dimension, we define metrics, which can have different types: *Foundational metrics* (FM) include raw, detailed data, often of a complex type. Examples include the complete records of an event’s peer review or the map of all persons involved in an event’s organization and their respective roles. *Estimated metrics* (EM) help to estimate the values of foundational metrics when the full raw data is not available. For example, the organizers of an event might not want to reveal the exact amount of a sponsor’s financial contribution for confidentiality, but they might want to publicly announce that it was a “platinum” sponsor, and that, for this event, this category started at €10,000. From a complex foundational metric, one can usually derive several more straightforward metrics that we call *derived metrics* (DM). This derivation often involves *aggregate functions* such as count, sum or minimum,^12^ as well as more complex arithmetics. For example, the acceptance rate can be derived from the full review records by aggregation.

Some metrics are, from a formal, ontological perspective, derived from foundational ones, but, in practice, more readily available than the latter. For example, the full review records of an event (a foundational metric) are typically not publicly available, whereas the acceptance rate derived from them is published. There are also metrics that we could, in principle, derived from publicly available data, such as the h-index of a person from freely accessible citation indexes. However, we nevertheless treat them as if they were foundational metrics, for two reasons: the derived value is readily available or deriving the respective metric would go beyond the scope of assessing the quality of an *event*, not to mention the computational resources it would require.

## Quality metrics

The core criteria of event quality are grouped into three categories: event-related, person-related and bibliographic metrics. Table 1 gives an overview on all dimensions and metrics. We provide detailed descriptions for some selected dimensions and metrics of each category in the sequel.

### Event-related

This section introduces metrics that directly represent event-related criteria.

#### Submissions

Researchers exchange their contributions in the shape of written documents following certain rules. Most events provide guidelines for writing and formatting documents to ensure consistency among submissions and fair competition among authors. These standards cover the layout of the submissions as well as their length (in pages), which may differ across submission types. The preferred style often follows standards established by the events’ publishers. In computer science, the most popular styles are the ACM, Springer LNCS, and IEEE styles (Yang and Davison 2012).

##### **Definition 1**

*Submissions* to an event must adhere to certain rules.

*Measuring* *FM1.1* is the set of all accepted submission styles. *FM1.2* is the set of accepted submission source formats, such as LaTeX, MS Word, ePUB or RASH (Research Articles in Simplified HTML, a subset of HTML Peroni et al. 2017). The length of the different types of submissions should not exceed a certain number of pages. An event may accept different types of submissions; *FM1.3* is the set of these types, e.g., {“full paper”, “short paper”, “poster”, “demo”}. Metric *FM1.4* maps submission types to their maximum length in pages, e.g., $\{ \text {``full paper''} \mapsto 16, \text {``demo''} \mapsto 8 \}$.

From these base metrics one can define a derived metric *DM1.5* for the overall flexibility of the event’s submission process, defined as the number of possible combinations of different submission styles, source formats and types, i.e. $DM1.5 :=\# FM1.1 \times \# FM1.2 \times \# FM1.3$. This helps to distinguish events restricted with regard to accepted styles, formats, and submission types from the more flexible ones. The license under which the publications of an event are available is represented by *FM1.6*. It is the extent to which the terms and conditions specified by the event organizers or publishers grant permission to access content legally. The possible values for this metric are considered as any combination of reuse conditions, e.g., attribution (BY), non-commercial (NC), share-alike (SA), and no derivative (ND), showing the copyright (by the publisher or the event) or open access.

*Advantages* A wide range of accepted submission formats may encourage a high number of submissions. If an event accepts papers of a length similar to that of a draft that a researcher has written already (e.g., submitted to an earlier event where it was rejected), then he or she can resubmit it to the new event with little extra effort.

*Disadvantages* An event’s commitment to one widely used style (e.g., LNCS but not ACM) does not permit conclusions about the event’s quality. Authors might refuse to submit to an event because of format restrictions. The most widely used format differs across disciplines; this can limit interdisciplinary cooperation.

#### Location

One of the crucial factors in holding a successful event is to select a suitable location, which attracts many participants and enables them to interact with each other conveniently.

##### **Definition 2**

An event is held in a geographical *location*.

*Measuring* We define a foundational location metric *FM2.1* that it presented as the triple $( City , Country , Continent )$. By extending this metric to event series, one can derive the number of distinct locations visited by an event (*DM2.2*). Another derived metric, *DM2.3*, maps every distinct location to the number of times the event has taken place there (by city, country, or continent). *DM2.4* takes the possible values “split”, “merge” or “keep”, indicating whether the previous edition of the event split into more than one successor event, merged with other events to form a broader successor event, or was kept as is.

*Advantages* Diversity in the location of an event increases the awareness of researchers about the existence of the event and its covered topics.

*Disadvantages* Holding events in high-priced and luxurious places, e.g., Hawaii for VLDB 2015 and all of the HICSS series, may discourage researchers with a low budget to register; on the other hand, high-profile researchers often either have a generous budget available or compete successfully for travel grants.

#### Review process

Reviewers play a central role in quality control within scholarly communities. They are experienced researchers from the same community and thus also called *peer reviewers* (What is peer review 2016). A reviewer is expected to comment on a submission, recommend its acceptance or rejection, and to provide a detailed justification of their decision with regard to criteria such as the originality and soundness of the research, the quality of the presentation, the relevance to the event, etc.

##### **Definition 3**

A *review process* is a series of rigorous decision-making activities, in which program chairs assign submissions to reviewers, who then comment and rate it, thus informing the program chairs’ decision on acceptance versus rejection.

*Measuring* Metric *FM3.1* indicates whether a formal review process exists at all. Metric *FM3.2* classifies the type of review process into two categories: having submissions reviewed by assigned peer reviewers, open community involvement, or both. Metric *FM3.3* indicates the type of review process: open, (single-)blind, or double-blind. Open review means that authors and reviewers know each other’s identities. In a single-blind review, the names of the reviewers are hidden from the author. Double-blind review means that neither authors nor reviewers know each other’s identities. Public peer review (PPR), or open peer review, leads to high-quality reviews and an elaborated peer review process (Bornmann et al. 2012).

*Advantages* Despite criticism, peer review is still the only widely accepted method for validating research (What is peer review 2016). Good reviews are increasingly encouraged and honored; a small but increasing number of conferences offers *best reviewer awards*. For example, the Editors of Labour Economics journal offer the Best Reviewer Award of €1000 for the best reviewer out of a list of 10 top reviewers in 2019.

*Disadvantages* No standard system for assessing the quality of peer reviews is generally in use.

#### Review results

Authors of submissions accepted in the review process are asked to improve them based on the reviewers’ feedback before they are published. Authors of rejected submissions typically also improve them and submit them to some other venue.

##### **Definition 4**

*Review results* comprise all information that the reviewers provide to the program chairs (who forward most of it to the authors), plus possibly additional information that the program chairs provide to the authors.

*Measuring* The foundational metric *FM4.1* refers to the full records of the review process (which are rarely publicly available), i.e., for each submission, the chairs’ final decision and a set of reviews authored by peer reviewers, where each review comprises at least the review text and an overall recommendation (e.g., “accept” or “reject”), in many cases also a confidential message to the program committee and multiple sub-scores (e.g., for relevance with regard to the topic of the conference, or for quality of presentation). The derived metric *DM4.2* indicates the minimum number of reviews per submission regardless of being accepted or rejected. Metric *DM4.3* measures the average length of reviews by characters of text. Reviews of less than ten lines of up to 80 characters would typically be considered insufficient. In most review forms, reviewers are asked to indicate their confidence about the topic covered by the submission as well as the relevance of submission to the event. Metric *DM4.4* measures the average confidence of all reviewers of an event. Metric *DM4.5* measures the ratio of reviews that the original assignee delegated to sub-reviewers. In such cases, there is the risk that the original assignee does not do justice to their responsibility to deliver high-quality reviews, e.g., when delegating to inexperienced reviewers and not guiding them properly. Metric *DM4.6* represents the average relevance of submissions indicated by reviewers. The average number of reviews per submission is depicted by *DM4.7*. The acceptance rate *DM4.8* is the ratio of submissions accepted after review. The overall composite score *DM4.9* is the average of all review scores, rounded to the nearest whole number.

*Advantages* Good quality reviews make an event an attractive submission target despite a low acceptance rate.

*Disadvantages* As reviews are not always written by experts, a high number of reviews or long review texts do not necessarily imply high-quality feedback. A low acceptance rate only reliably indicates a good quality of accepted submissions when there are many strong submissions.

#### Publishing

Long-lasting, trustworthy organizations, i.e., publishers, publish archival scientific publications. Persistent identifiers such as DOIs or ISBNs are used to uniquely identify archival publications. It is often mentioned as a rule of thumb that the best conferences are supported by well-known publishers such as, in computer science, ACM, IEEE or Springer (Ernst 2006). Even medium or beginner events involving a prestigious publisher in the publishing process can be considered good events, as they have passed a quality-based admission process defined by the publisher. For example, the proceedings of the SAVE-SD workshop co-located with the WWW conference are published in Springer’s LNCS series, which puts the workshop on par with many well-reputed computer science conferences.

##### **Definition 5**

*Publishing* is the act of disseminating an event’s *proceedings*, which include the final versions of all accepted submissions. The publisher is a commercial or non-profit *organization*.

*Measuring* Metric *FM5.1* depicts the set of names of all publishers involved in a super-event as well as its co-located sub-events. *DM5.2* indicates the existence of an official publisher. In each community, publishers have a certain popularity level. In *DM5.3* the popularity of a publisher is represented. Popularity can be measured by inviting experts of the community to create a subjective list of publishers whose involvement would motivate them to submit to an event.

*Advantages* Reputation of publishers influences the reputation of events.

*Disadvantages* One cannot clearly say whether having had several publishers throughout its history is a plus for an event series or not. It is not an easy task to measure the reputation of a publisher.

#### Journal$\leftrightarrow$event coupling

Although events have originally had an entirely different focus compared to journals, these boundaries increasingly blur. Meanwhile, there are various methods established how events and journals can be coupled, the most important ones being:

*Loose coupling* The review and selection processes of the event and the journal are entirely separated. However, best-ranked submissions in the review of the event are invited either to a special issue or as a regular journal submission. Still, a significant extension of the invited articles is commonly requested, and a completely independent peer-review process is usually performed by the journal (to which prior reviews might be made available or not). Conversely, selected journal publications might be presented at the event on a special track or as part of the regular program. In the latter case, a selection is performed by the event’s PC or the journal editors, but rarely an additional, full-fledged separate review process is performed.

*Close coupling* A certain percentage of accepted publications at the event is automatically accepted to a journal. Extensions to the original publications might be required, and another peer-review cycle might be performed.

*Full coupling* There is only a single review process for both the event and the journal. All accepted submissions will be presented both as articles in the journal and as presentations at the event. Hence, the journal serves as a publishing outlet for accepted conference submissions or, in other words, the conference serves as a presentation venue for accepted journal publications. Since there is only one type of submission and a single review process, there is no risk of journal articles only being marginal extensions of the former conference papers. An example of full coupling is the *Conference on Very Large Databases* (VLDB), which has been following a journal-style peer-review and publishing process since 2008 (Jagadish 2008).

##### **Definition 6**

*Journal*$\leftrightarrow$*event coupling* refers to a defined method of combining the review and publishing process of one or multiple events with one or multiple journals.

*Measuring* *FM6.1 Coupling type*—indicates the type of journal$\leftrightarrow$event coupling, i.e., loose (accepted papers are invited for submission to the journal or presentation at the event), close (fast, aligned review track for accepted papers at the event) or full (a single review process and submission type). The other five metrics are *FM6.2* Journal name, *FM6.3* Journal publisher, *FM6.4* Journal popularity, *FM6.5* Eigenfactor Score, *FM6.6* Journal impact factor. The Article Influence Score *FM6.7* is similar to the Eigenfactor Score; the only difference is that it has an additional normalization to the number of published papers. It shows the average influence of a journal’s articles for the first 5 years after publication.

*Advantages* A journal$\leftrightarrow$event coupling is usually attractive for authors, since, in the loose and close coupling types, one research work is published twice (in different stages), but with some alignment of the peer-review processes and thus reduced improvement, revision, and communication effort. If the journal peer-review process is properly implemented and has a particular focus on evaluating the substantial extension and maturation of the original work (presented at the conference), a coupling can help to make the research-review-publication life-cycle more effective and efficient. The full journal$\leftrightarrow$event coupling actually combines the best of both worlds: accepted submissions can be assumed to have gone through a thorough, selective and multi-stage peer-review process while being presented at an event, thus facilitating the discussion with a larger audience (cf. Jagadish 2008 for a detailed discussion).

*Disadvantages* If not properly implemented, the loose or close coupling is prone to result only in marginal extensions and thus two publications (event and journal) with marginal differences. In loose or close coupling, both event and journal might suffer from reduced bibliometric impact, since attracted citations have to be shared between both publications if their difference is marginal. A loose or close journal-event coupling not implemented properly might result in a wrong incentivization, in that authors are encouraged to maximally exploit publication output while minimizing the research effort.

#### Discoverability

##### **Definition 7**

*Discoverability* is the extent to which interested parties can find events relevant to their research interests.

*Measuring* Two factors can directly influence the search results while searching the Web: the search engine and the set of search keywords. *DM7.1* measures the average rank of the targeted event in the list of results of searching for its full name, *DM7.2* by the acronym (with year for a single event, without a year for the series), and *DM7.3* by topic.

*Advantages* Discoverability increases users’ awareness of the existence of relevant conferences. When a relevant conference is easily discoverable, it saves researchers much time.

*Disadvantages* Researchers might miss the chance of submitting and getting accepted in a good event because of not being aware of it or being late to discover it. An event that is not easily discoverable indirectly restricts the chance of paper submission, and consequently, acceptance. An event that is not easily discoverable influences its own quality and reputation.

#### Reputation and impact

Events have a particular reputation among the members of their communities. Hard evidence for reputation in terms of quality metrics is increasingly requested—which is precisely the motivation for our research. A full, rigorous assessment of an event’s reputation could be achieved by computing an appropriately weighted sum of all of its quality metrics. Where this is not feasible, one often resorts to measuring an event’s impact by counting the citations of its publications.

##### **Definition 8**

An event has a certain *Reputation* in the community, which can be subjective, or based on a rigorous assessment of the overall *Impact* of the event’s publications.

*Measuring* Jianying Zhou proposed a quantitative metric for the “conference impact factor” (CIF,^13^*FM8.1*) as follows:

$$\begin{aligned} CIF =\frac{1}{ AR + CR + PR } \end{aligned}$$

where

$$\begin{aligned} AR (={ DM6.6}) = \frac{\# accepted\ papers }{\# submissions },~~ CR = \frac{\# accepted\ papers }{\# h5 citations } ~and \end{aligned}$$

$$\begin{aligned} PR = \frac{\# accepted\ papers }{\# registered\ participants (={ FM2.1})} \end{aligned}$$

We treat this metric as “foundational” in its dimension. Its definition depends on multiple metrics for different dimensions. As a citation metric, it uses the h5-median as explained below. The CIF defined this way is high for events with many submissions, many citations and many registered participants in relation to the number of accepted papers.

Simple h-index estimation (SHINE)^14^ is a Brazilian website that provides h-index calculation for conferences. Google Scholar provides the h5-index for high-profile events: the h-index for articles that have been published in the last 5 years. Looking back from 2018, this index means that in the period 2013–2017, *h* publications have been cited at least *h* times each over these years. We define *FM8.2* as “the h5-index as provided by Google Scholar”. Google defines the “h5-median”, which is used to define the CIF (*FM8.1*), for a publication as “the median number of citations for the articles that make up its h5-index”.^15^

We can consider conference rankings as a further metric. As a concrete example, we define as *FM8.3* the CORE ranking. Their system classifies conferences in four categories: A* (exceptional) conferences are leading events in a discipline area, A (excellent) conferences are highly important events in a discipline area, B (good) conferences are well regarded in a discipline area, and C (satisfactory) are those events that meet minimum standards.

*Advantages* Currently, the most reliable metric to decide to which extent a scientific event has high quality is the acceptance rate because it is widely available, calculated transparently, and hard to manipulate.

*Disadvantages* It is not easy to find out the acceptance rate of events that do not publish this information. The acceptance rate can vary among different editions of the same event series. It takes time for a new series to arrive at a stable acceptance rate. Interpreting the acceptance rate without taking into account the capacity of an event does not necessarily lead to reliable conclusions; thus the more complex CIF definition. In contrast to certain other metrics, the acceptance rate of a new event is unknown before it has first taken place. The h-index of an event series may not be reliable since series that have existed for many decades with thousands of papers will typically rank higher than recent series that accept only a small number of papers.^16^

#### Sponsorship

Any organization providing financial funding for an event is known as a sponsor. Sponsors are typically classified into levels depending on the amount of their financial support. For example, for the WWW 2015 conference the levels were: Gold (€30,000), Silver (€20,000+), Bronze (€10,000+), and supporter with less or no financial support but offering help, e.g., with video recording. Different benefits for sponsors are offered in the sponsorship packages by event organizers. Those sponsorship benefits that include the presence of sponsors in the event as an exhibition or standing in special sessions are related to other metrics such as event program, number, and length of breaks and sessions.

##### **Definition 9**

*Sponsorship* means that external organizations support an event financially.

*Measuring* The exact distribution of financial support provided by all sponsors (*FM9.1*) is defined as $S_ exact :=\{(s_1,c(s_1)),\ldots ,(s_n,c(s_n))\}$; where $\{s_1,\dots ,s_n\}$ are the sponsors, and $c(s_i)$ denotes the exact amount of financial support provided by sponsor $s_i$. *FM9.1* is the most comprehensive sponsorship metric, from which all derived metrics can be computed. In practice, the exact amount of financial support is rarely published, but the level of sponsorship (e.g., silver) is. *FM9.2* associates with each sponsor of an event its level (as a string). The values of this metric are not comparable across events, not even necessarily within the same series: different events might define different thresholds for becoming, e.g., a gold sponsor, they might not make use of all levels. However, combining *FM9.2* with the definitions of sponsorship levels of the respective events enables an estimation of *FM9.1* via an estimated metric *EM9.3*, which associates to each sponsor the *range* in which its financial support is, e.g., the interval $[\$500,\$1000)$ for a bronze sponsor or [€$5000,\infty )$ for a platinum sponsor.

The total number of sponsors of an event *DM9.4* can be derived from each of the metrics *9.1* to *9.3* in a straightforward way. *DM9.5*, the exact sum of sponsors’ financial contributions, can be computed from *FM9.1* as $\sum _{i=1}^n c( s _i)$. When the exact contributions are not known, one can compute the estimated amount of overall sponsoring (*DM9.6*) from *EM9.3* by using the lower bounds of the intervals that correspond to the sponsoring levels.

The reputation of sponsors *DM9.4* is a set of attributes that can be derived from the past performance of an organization. Goldberg et al. propose a ranking method in the range of “poor” to “good”, which does, however, not give reasons why one firm has a better or poorer reputation than another one (Goldberg and Hartwick 1990). As one attribute to measure the reputation of the sponsors, we look into the list of 1000 fortune and admired companies worldwide, which is published every year *Y* (= 2015, year of assessment and year of held events) (Morais 2015).

By *DM9.5* we classify sponsors as local or global by comparing the location of the event with that of the sponsor organizations. We measure continuity of sponsors (*DM9.6*) by looking at the list of organizations that have appeared as sponsors for the events in a series.

*Advantages* Sponsors help ensure an event to be better to sustain an event over time. Sponsors with a high reputation may attract a broader audience and highly reputed people. Measuring this dimension helps to find out how competitive an event is. A long and continuous sponsorship history indicates that the sponsor has been well maintained in the past. Having many high-level sponsors an event has, the more financial support it has.

*Disadvantages* Reputation is an intangible and complex concept, may change over time.

#### Co-location

Co-Location means the fact of making a tenancy in common for related events. All events taking place at the same time in the same place are called co-located events, such as workshops co-located with conferences. When a co-located event is a sub-event of a super-event, their overall respective qualities influence each other in both directions.

##### **Definition 10**

*Co-Location* comprises super, sub, or sister events taking place in the same place and at the same time as the event whose quality is being assessed. This dimension addresses the *relationships* between co-located events, whereas the *quality* of a co-located event is assessed like that of any other event.

*Measuring* The co-located event metric *FM10.1* is the fundamental metric of this dimension: a list of co-located sub-events of a co-located super-event. The number of co-located events *DM10.2* is derived in a straightforward way. The *FM10.3* metric has a boolean value indicating whether admission criteria are required to organize a co-located event with a super event or not. As arbitrary types of events, such as conferences or workshops, may be co-located in arbitrary combinations, it is important to see whether a co-located event is a conference or a workshop. The types of co-located events are collected in *FM10.4*. The following three metrics are defined as aggregate values over the overall quality of each co-located event, computed as a weighted sum over its individual quality metrics: *DM10.5* quality of co-located sub-events, *DM10.6* quality of co-located super-events, and quality of co-located super-events, *DM10.7* quality of sister events. In practical computation, a mutually recursive definition of the quality of an event and of its co-located events should be avoided, e.g., when computing the quality of each co-located sub-event for the purpose of determining *DM10.5* of the super-event, *DM10.6*, which in turn depends on the super-event’s quality, should be skipped. Finally, metric *DM10.8* represents the reputation of co-located events.

*Advantages* Having more co-located events attracts a broader audience. Co-located events might influence/change/increase the research direction of the overall event. Information about co-located events may helps with filtering events, e.g., when listing all conferences on a given topic that have a PhD consortium.

*Disadvantages* It is difficult to distinguish the exact influence of co-located sub-events on each other and on the co-located super-event and the other way around. It is hard to distinguish tracks with a high level of autonomy (e.g., their own sub programme committees) from proper sub-events.

#### Topical focus

##### **Definition 11**

*Topical focus* refers to the research topics addressed by an event, and whether they are clearly defined, innovative, and recent.

*Measuring* Every event has a title that typically makes it unique in the community *FM11.1*. There are often fake conferences, or non-fake but new conferences that intentionally use titles that look similar to established ones. The focus type *DM11.2* can be defined as narrow, medium, and high by locating it in a hierarchical classification scheme such as the ACM Computing Classification System.^17^ The focus of an event is affected by concept drift and other notions shifting over time (cf. Osborne and Motta 2014). Concept drift can be observed from comparing different versions of classification schemes, e.g., the ACM CCS of 1998 and 2012. *DM11.3* represents the coverage of innovative and recent topics in the area of interest.

*Advantages* From analyzing the topics focus of events, one can observe the historical development and emergence of research topics, communities and corresponding events.

*Disadvantages* Text mining approaches are required for a high quality of assessments.

#### Registration

##### **Definition 12**

*Registration*, which involves the payment of a fee in most cases, is a prerequisite for participating in most scientific events.

*Measuring* *FM12.1* comprises the amount of registration fees per category (e.g., “full, non-student participant, late registration”) for an event. Each event has a registration method, which influences the ease of the registration process, which we consider in metric *DM12.2*. *DM12.3* indicates whether the event includes any kind of student discount, which makes it easier for students to participate.

*Advantages* Low registration fees and an easy registration procedure encourage researchers with a low budget to participate.

*Disadvantages* Good quality events usually have high registration fees. High registration fees discourage researchers from participating in events.

#### Schedule

One of the challenging tasks for the event organizers is to build a good schedule for the event, even with the assistance of software.

##### **Definition 13**

The *Schedule* of an event comprises the presentations of its accepted submissions, as well as invited presentations, panels, networking events, breaks, etc.

*Measuring* We look into four metrics to measure how good the schedule of an event is. *FM13.1*, as the primary metric, takes the full schedule of the event. In metric *DM13.2*, we sum up the lengths of breaks in minutes, while *DM13.3* counts the number of breaks per day. Event duration is represented by *DM13.4*. The number of sessions per day is depicted by metric *DM13.5*, and the number of presentations per session is measured by *DM13.5*. The order of the presentations and sessions may also influence the overall quality of the schedule, but such considerations are out of scope of our current study.

*Advantages* A good schedule can improve the attention and creativity of participants and increase the chance of meeting more people in an event rather than only taking part in presentations. Furthermore, research shows that taking a break after learning something helps in long term memorizing and increases the creativity of people (Tambini et al. 2010; Little et al. 2017).

#### Awards

Events often announce awards that could be given to participants, mainly to authors.

##### **Definition 14**

*Awards* are offered to participants of an event to honor outstanding efforts.

*Measuring* Awards of an event vary depending on the motivation of an events’ organizers, the intended purpose and the research area. For example, an event with a topic focus on content-based search and document analysis usually offer awards for the objectively best performance in the sense of information retrieval metrics, whereas other events could offer an award for the subjectively most innovative approach. In metric *DM14.1*, types of awards have been determined. For financial awards, the amount of award is measured in *DM14.2*.

*Advantages* Besides h-index and several other metrics, having a list of awards in one’s curriculum vitae influences the evaluation of the academic achievements of researchers.

#### Publicity

Kotler defines publicity as stimulation of demand for any service by placing important news about it in public media (Kotler 2000). Social media make this task easy and free of charge for event organizers.

##### **Definition 15**

*Publicity* refers to announcing an event within the community and beyond, via diverse communication channels.

*Measuring* An event’s chairs—often there is a publicity chair—use different communication channels *FM15.1* to make announcements about the event. The primary option is the homepage of the event, but it can also be the existing mailing lists in the area of interest, or social networks. This is not a direct indicator influencing the quality of an event, but good publicity can increase the awareness about the event and may thus attract submissions and participants. *FM15.2* measures the usability of an event’s homepage, taking into account established usability and accessibility standards, e.g., whether it is mobile-friendly. The metric *FM15.3* measures the homepage’s usefulness, e.g., its comprehensiveness w.r.t. the amount of information provided. For general definitions of usability and usefulness in the area of digital libraries, see, e.g., Fuhr et al. (2007).

*Advantages* Like advertising, awareness about the event can be increased by publicity.

### Person-related

Person related metrics aim at measuring the extent to which the persons involved in an event have an impact on its quality.

#### Organizers and roles

People involved in various ways are essential for events because they provide inspiration, creativity, vision, and motivation. They provide the skills, competencies, and labor necessary to make an event work.

##### **Definition 16**

*Organizers and Roles* in the broad sense include all people involved in an event in different roles, regardless of physical presence.

The responsibilities of people involved in different roles depend on the particular event’s structure and size. Large first-tier conferences, such as TheWeb conference, have a four-layer program committee structure consisting of program chairs, track chairs, senior PC members, and ordinary PC members. Smaller events lack the track and/or senior PC chair layer.

*Measuring* People are involved in events with different roles. *FM16.1* is a function that maps the identity of a persons to the set of that person’s roles. Roles in a scientific event include general chair, program committee chair/senior member/member, local organizer, author, keynote/invited speaker, and participant. Person reputation *DM16.2* is the extent to which a person involved in a scientific event is popular and active in the community. Reputation can be measured by a number of indicators, a detailed discussion of which is out of scope here. For academics, these can be, for example, number of publications, number of citations, h-index, i10-index, etc. The frequency of involvement *DM16.3* is a metric derived from *FM16.1*, which shows the number of times a person has held a role in a particular event series. The frequency of same-role involvement *DM16.4* is a metric that shows the number of times a person has had the same role in an event series.

*Advantages* The involvement of high-profile researchers in an event can improve the quality and raise enthusiasm among prospective authors and attendees for an event.

#### Person backgrounds

Events typically aim at attracting diverse participants.

##### **Definition 17**

*Participant Background* includes characteristics of participants such as experience or workplace.

*Measuring* The participants’ experience level *DM17.1* can be measured by computing their average number of years of experience. *DM17.2* determines the countries that persons come from, defined as the country in which the organization is located that they are affiliated with. *DM17.3* associates persons to sectors, such as industry or academia.

*Advantages* Event participants with a diversity of experience levels have an impact on other dimensions, e.g., topic coverage, ways of publicity, good quality reviews, etc. People involved from different countries increase the awareness about the event and its impact.

**Table 1:** Overview of quality of quality for person and bibliographic data related dimensions and of the characteristics of their metrics (✗ means that the criterion applies to the metric, $\checkmark$ means that it does not apply; type = S means subjective, O means objective)

Dimension	Metric	Type of Impl.	Ease of collect	Data avail.	Data reliab.	Data precision	Ease of comput.
*M1. Submissions*	FM1.1 Accepted styles	–	$\checkmark$	$\checkmark$	$\checkmark$	$\checkmark$	$\checkmark$
	FM1.2 Accepted formats	–	$\checkmark$	$\checkmark$	$\checkmark$	$\checkmark$	$\checkmark$
	FM1.3 Submission types	*(prop)*	$\checkmark$	$\checkmark$	$\checkmark$	$\checkmark$	$\checkmark$
	FM1.4 Page count	–	$\checkmark$	$\checkmark$	$\checkmark$	$\checkmark$	$\checkmark$
	DM1.5 Submission combinations	–	$\checkmark$	$\checkmark$	$\checkmark$	$\checkmark$	$\checkmark$
	FM1.6 License	–	$\checkmark$	$\checkmark$	$\checkmark$	✗	✗
*M2. Location*	FM2.1 Location	*(cate)*	$\checkmark$	$\checkmark$	$\checkmark$	$\checkmark$	$\checkmark$
	DM2.2 Location-visited	*(temp)*	$\checkmark$	$\checkmark$	$\checkmark$	$\checkmark$	✗
	DM2.3 Distribution of locations	*(ASK)*	✗	$\checkmark$	$\checkmark$	$\checkmark$	✗
	DM2.4 Event type (split/merge)	*(temp)*	✗	$\checkmark$	$\checkmark$	✗	✗
*M3. Review process*	FM3.1 Existence of review process	–	$\checkmark$	$\checkmark$	$\checkmark$	$\checkmark$	$\checkmark$
	DM3.2 Review type	–	$\checkmark$	$\checkmark$	✗	$\checkmark$	$\checkmark$
	FM3.3 Reviews process type	–	$\checkmark$	$\checkmark$	✗	$\checkmark$	$\checkmark$
*M4. Review results*	FM4.1 Full review records	–	✗	✗	✗	✗	✗
	DM4.2 Minimum number of reviews	–	✗	✗	✗	✗	✗
	DM4.3 Avg. length of reviews	–	✗	✗	✗	✗	✗
	DM4.4 Avg. confidence of reviewers	–	✗	✗	✗	✗	✗
	DM4.5 Delegation ratio	–	✗	✗	✗	✗	✗
	DM4.6 Avg. relevance of submissions	–	✗	✗	✗	✗	✗
	DM4.7 Avg. no. of reviews	–	✗	$\checkmark$	✗	✗	✗
	DM4.8 Acceptance rate	*(temp)*	✗	✗	✗	✗	✗
	DM4.9 Overall composite score	*(temp)*	✗	✗	✗	✗	✗
*M5. Publishing*	FM5.1 List of publishers	*(prop)*	$\checkmark$	$\checkmark$	$\checkmark$	$\checkmark$	$\checkmark$
	DM5.2 Existence of official publisher	–	$\checkmark$	$\checkmark$	$\checkmark$	$\checkmark$	$\checkmark$
	DM5.3 Publisher popularity	*(prop)*	✗	✗	✗	✗	✗
*M6. Journal*$\leftrightarrow$*event coupling*	FM6.1 Coupling type	–	$\checkmark$	$\checkmark$	$\checkmark$	$\checkmark$	$\checkmark$
	FM6.2 Journal name	*(prop)*	$\checkmark$	$\checkmark$	$\checkmark$	$\checkmark$	$\checkmark$
	FM6.3 Journal publisher	*(prop)*	$\checkmark$	$\checkmark$	$\checkmark$	$\checkmark$	$\checkmark$
	FM6.4 Journal popularity	–	✗	✗	✗	✗	✗
	FM6.5 Eigenfactor score	–	✗	✗	✗	✗	✗
	FM6.6 Journal impact factor	*(prop)*	✗	✗	✗	✗	✗
	FM6.7 Article Influence Score	*(prop)*	✗	✗	✗	✗	✗
*M7. Discover-ability*	DM7.1 By full name	*(prop)*	✗	✗	✗	✗	✗
	DM7.2 By acronym	*(prop)*	✗	✗	✗	✗	✗
	DM7.3 By topic	*(cate)*	✗	✗	✗	✗	✗
*M8. Reputation and Impact*	FM8.1 Impact factor	–	✗	$\checkmark$	$\checkmark$	$\checkmark$	$\checkmark$
	FM8.2 h5-index	*(prop)*	$\checkmark$	$\checkmark$	$\checkmark$	$\checkmark$	$\checkmark$
	FM8.3 Rank	*(prop)*	$\checkmark$	$\checkmark$	$\checkmark$	$\checkmark$	$\checkmark$
*M9. Sponsorship*	FM9.1 Distribution of finance cont.	–	✗	$\checkmark$	$\checkmark$	✗	✗
	EM9.2 Distribution of sponsors/type	*(ASK)*	✗	$\checkmark$	$\checkmark$	✗	✗
	DM9.3 No. of sponsors	–	$\checkmark$	$\checkmark$	$\checkmark$	$\checkmark$	✗
	DM9.4 Reputation of sponsors	–	✗	$\checkmark$	$\checkmark$	$\checkmark$	✗
	DM9.5 Type of sponsor local/global	*(prop)*	✗	$\checkmark$	$\checkmark$	$\checkmark$	✗
	DM9.6 Continuity of sponsors	*(SPARQL)*	✗	$\checkmark$	$\checkmark$	$\checkmark$	✗
*M10. Co-Location*	FM10.1 Co-Located events	*(temp)*	$\checkmark$	$\checkmark$	$\checkmark$	$\checkmark$	$\checkmark$
	DM10.2 No. of co-located events	*(prop)*	$\checkmark$	$\checkmark$	$\checkmark$	$\checkmark$	$\checkmark$
	FM10.3 Admission criteria:	–	$\checkmark$	$\checkmark$	$\checkmark$	$\checkmark$	$\checkmark$
	FM10.4 Type of co-located events	*(prop)*	$\checkmark$	$\checkmark$	$\checkmark$	$\checkmark$	$\checkmark$
	DM10.5 Qual. of co-loc. sub-events	–	✗	$\checkmark$	$\checkmark$	$\checkmark$	$\checkmark$
	DM10.6 Qual. of co-loc. super-events	–	✗	$\checkmark$	$\checkmark$	$\checkmark$	$\checkmark$
	DM10.7 Qual. of sister events	–	✗	$\checkmark$	$\checkmark$	$\checkmark$	$\checkmark$
	DM10.8 Repu. of co-located event	–	✗	$\checkmark$	$\checkmark$	$\checkmark$	$\checkmark$
*M11. Topical focus*	FM11.1 Event name	*(prop)*	$\checkmark$	$\checkmark$	$\checkmark$	$\checkmark$	$\checkmark$
	FM11.2 Focus type	*(cate)*	$\checkmark$	$\checkmark$	$\checkmark$	$\checkmark$	$\checkmark$
	FM11.3 Coverage of innovative topics	*(cate)*	$\checkmark$	$\checkmark$	$\checkmark$	✗	$\checkmark$
*M12. Registration*	FM12.1 Fees	*(prop)*	$\checkmark$	$\checkmark$	$\checkmark$	$\checkmark$	$\checkmark$
	DM12.2 Ease of registration	–	✗	✗	$\checkmark$	$\checkmark$	$\checkmark$
	DM12.3 Student discounts	*(prop)*	$\checkmark$	$\checkmark$	$\checkmark$	$\checkmark$	$\checkmark$
*M13. Schedule*	FM13.1 Full schedule	*(temp)*	$\checkmark$	$\checkmark$	$\checkmark$	✗	$\checkmark$
	DM13.2 Avg. length of breaks	*(SPARQL)*	$\checkmark$	$\checkmark$	$\checkmark$	✗	$\checkmark$
	DM13.3 No. of breaks	*(prop)*	$\checkmark$	$\checkmark$	$\checkmark$	$\checkmark$	$\checkmark$
	DM13.4 Avg. event duration	*(temp)*	$\checkmark$	$\checkmark$	$\checkmark$	$\checkmark$	$\checkmark$
	DM13.5 No. of sessions per day	*(temp)*	$\checkmark$	$\checkmark$	$\checkmark$	$\checkmark$	$\checkmark$
	DM13.6 No. Presentations per session	*(SPARQL)*	$\checkmark$	$\checkmark$	$\checkmark$	$\checkmark$	$\checkmark$
*M14. Awards*	DM14.1 Type of awards	*(prop)*	$\checkmark$	$\checkmark$	$\checkmark$	$\checkmark$	✗
	DM14.2 Amount of awards	*(prop)*	$\checkmark$	✗	$\checkmark$	✗	$\checkmark$
*M15. Publicity*	FM15.1 Communication channels	*(prop)*	✗	✗	✗	✗	✗
	FM15.2 Homepage usability	–	$\checkmark$	$\checkmark$	$\checkmark$	✗	$\checkmark$
	FM15.3 Homepage usefulness	–	$\checkmark$	$\checkmark$	$\checkmark$	✗	$\checkmark$
*M16. Organizers and Roles*	FM16.1 Person Roles	*(prop)*	$\checkmark$	$\checkmark$	$\checkmark$	✗	✗
	DM16.2 Person reputation	*(prop)*	✗	✗	$\checkmark$	✗	✗
	DM16.3 Freq. of involvement	*(SPARQL)*	$\checkmark$	✗	$\checkmark$	✗	✗
	DM16.4 Freq. of same-role	*(SPARQL)*	✗	✗	$\checkmark$	✗	✗
*M17. Person Backgrounds*	DM17.1 Experience level	–	✗	✗	✗	✗	✗
	DM17.2 Countries	*(ASK)*	✗	✗	✗	✗	✗
	DM17.3 Sectors	–	✗	✗	✗	✗	✗
*M18. Publication Avail. & Access.*	FM18.1 Indexing	–	$\checkmark$	$\checkmark$	$\checkmark$	✗	✗
	FM18.2 Accessibility	–	$\checkmark$	$\checkmark$	$\checkmark$	✗	✗
*M19. Biblio-metrics*	FM19.1 No. of publications	*(prop)*	$\checkmark$	$\checkmark$	$\checkmark$	$\checkmark$	✗
	FM19.2 No. of citations per publication	*(SPARQL)*	✗	$\checkmark$	$\checkmark$	✗	✗
	FM19.3 Avg. citations per publication	–	✗	$\checkmark$	$\checkmark$	$\checkmark$	$\checkmark$
*M20. Social network impact*	FM20.1 No. Page view	–	✗	$\checkmark$	$\checkmark$	✗	✗
	FM20.2 No. Discussed	–	✗	✗	✗	✗	✗
	FM20.3 No. Twitter hashtags	*(SPARQL)*	✗	$\checkmark$	$\checkmark$	✗	$\checkmark$
	FM20.4 No. Recommended	–	✗	✗	✗	✗	✗

### Bibliographic-related

The outcome of a positive review is a set of accepted submissions, which will be published (cf. section “Publishing”) and presented in the event.

#### Publication availability and accessibility

##### **Definition 18**

*Availability and Accessibility of Publications* of an event refers to its metadata as well as its full texts.

*Measuring* *FM18.1* measures the indexing of the individual publications of an event (actually of the *metadata* of its publications) in indexes such as the commercial Web of Science, Scopus, or the free DBLP. Accessibility of full texts is considered in *FM18.2*. We measure whether the publications of an event are published in institutional open access repositories or global ones such as Zenodo^18^ or arXiv.^19^

*Advantages* Easy availability and accessibility of publications of earlier events may give potential participants an impression of the topics covered and of the level of quality expected of an accepted contribution.

#### Bibliometrics

##### **Definition 19**

*Bibliometrics* is the statistical analysis of the publications of an event.

*Measuring* The number of publications, i.e., accepted papers (*FM19.1*), shows to what extent the event is productive. In order to assess the impact of an event, the number of citations per publication (*FM19.2*) is studied. *FM19.3* measures average citations received per article published in a series of events, and it is calculated by total citation count divided by the total publication count.

*Advantages* The quality and quantity of publications and number of citations provide a key measure of the event productivity. Bibliometrics of an event indicates the extent to which the event helped researchers and their contributions to be recognized in the community.

*Disadvantages* The number of citations alone is not a sufficient quality indicator; citations should be deeply analyzed (cf. the different types of citations classified by Peroni and Shotton (2012) and the discussion in section “Introduction”).

#### Social network impact

##### **Definition 20**

*Altmetrics* is considered as a composite indicator representing the *impact* of the event with regard to publicity of its publications in “alternative” ways.

We adopt the Altmetrics definition by ImpactStory and Public Library of Science and define the following metrics: *FM20.1* represents the number of HTML page views of the publications and the number of times the publications have been downloaded, *FM20.2* shows the number of times the papers published by the event have been discussed in social media such as Twitter, Facebook, or science blogs and the number of times the papers are saved in social bookmarking systems. *FM20.3* counts the number of Twitter hashtags corresponding to the event, and *FM20.4* represents the number of times the publications of an event is recommended by peer review systems such as F1000Prime.^20^

*Advantages* The social network impact dimension addresses the publicity of an event and its individual publications and how outstanding an event is w.r.t. its audience.

*Disadvantages* Although these metrics could be objectively measured, it is hard to determine their precise values.

### Event history

Almost all of the event-related metrics defined in the previous section “Event-related” can be extended to event *series*, i.e., considering each edition of an event. Formally, a series metrics is defined as an aggregation over the values of single-event metrics. In the following, we present examples of where valuable insight can be obtained by computing such aggregates.

*Length of the history* can be defined as (1) the number of editions, or (2) the time difference between the most recent edition (alternatively: the current date) and the first edition of an event. This metric helps to assess how well an event is established in its community. Formally, this metric can be defined as the extension of two trivial metrics not explicitly introduced in section “Event-related”: (1) the (Boolean) fact whether an event took place, or, (2) the date of an event.

*Regularity* is defined as the average distance of any two successive events on the time axis, e.g., “1 year” for an event that takes place every year at the same time.

*Continuity* refers to how continuously an event series has been held over its history. We reuse the formula presented in Fathalla et al. (2017a) for continuity as $\textit{C}= \min \left\{ 100\%,(\textit{E}*\textit{R})/\textit{A}\right\}$ to calculate the percentage of continuity for a specific conference where *C* stands for continuity, *E* for the number of editions of the event, *R* for the regularity of the event editions (1 for “every year”, 2 for “every two years”), and *A* for the age, counting the number of years since the first time the event was established. The year is the granularity for this metric.

*Monotonicity* is relevant, e.g., when applied to the number of *submissions* or *participants*.

It is a boolean metric whose value is *true* if the underlying number has been growing from every edition of the event to the following one.

*Diversity* is defined as the degree of variation of one metric, e.g., the number of distinct countries in which the events of a series have taken place.

All of these metrics are defined in an objective way.

oVERALL, most of the metrics defined for individual events can be extended to series, except for the following ones: *DM2.2 Location-visited* (which actually is an extension of *FM2.1* to series), *DM2.3 Distribution of locations*, *DM2.4 Event type (split/merge)* (a non-standard aggregation over two series), *DM9.6 Continuity of sponsors* (which can be derived from *FM9.1* like continuity of occurrence as defined above), *DM16.3 Freq. of involvement*, and *DM16.4 Freq. of same-role involvement* (which are both already aggregates over person metrics).

### Dependency graph of dimensions

**Fig. 2:**
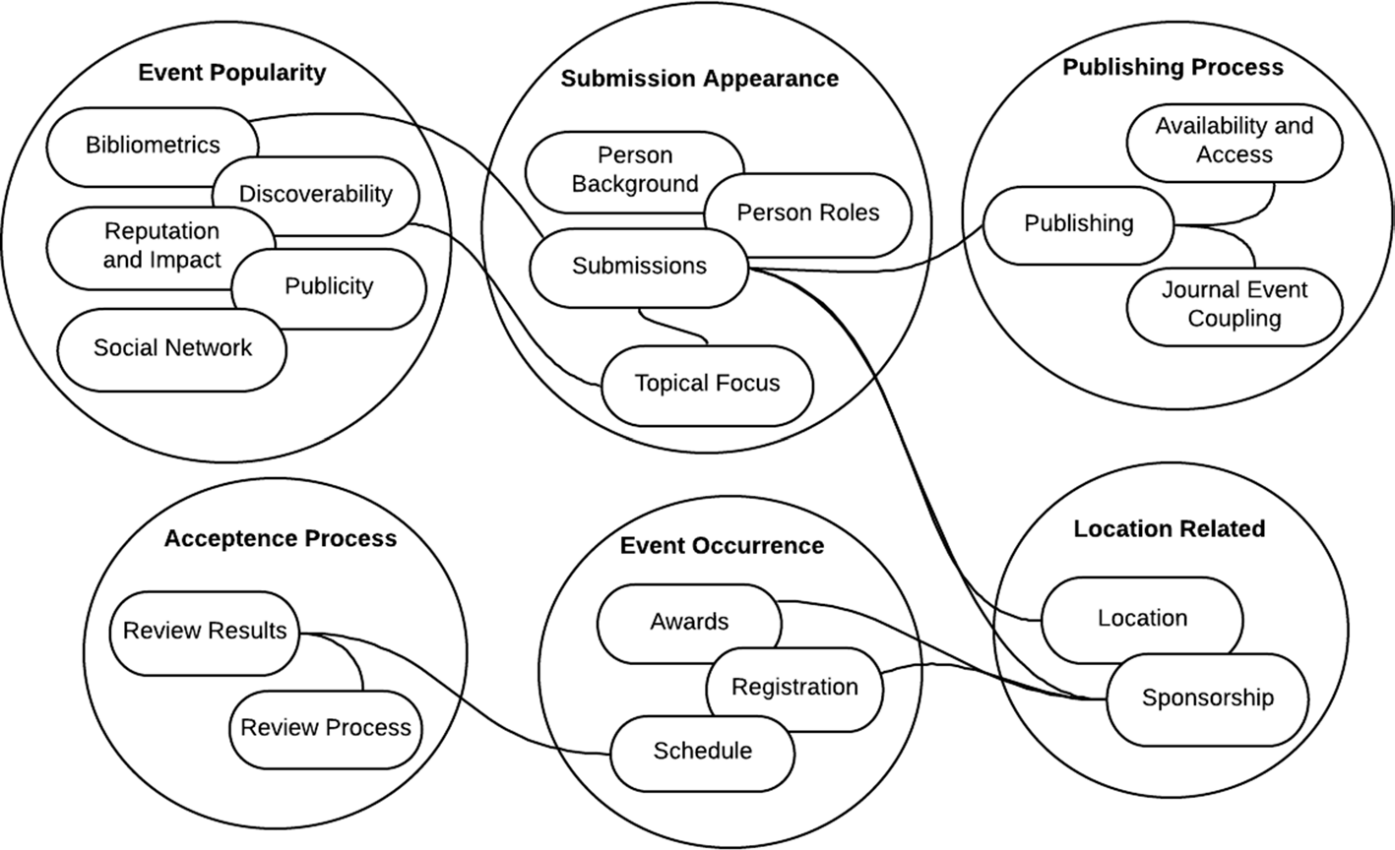
Dependency between dimensions

The metrics for quality assessment of scientific events are not independent of each other. A higher level dependency graph is shown in Fig. 2. For example, more sponsors may become active based on the location of the conference, or discoverability of an event that can affect its bibliometrics.

## Use cases for quality metrics

The ability to compute metrics, as defined in the previous section, can serve the scientific community in various ways. Instead of having oversimplified rankings for scientific events, quality metrics allow for developing a differentiated evaluation mechanism that takes individual preferences of researchers into account.

*Authors* For example, the derived event ranking can be combined with restrictions on fees (DM11.1) or the reputation of co-located events (DM3.8). Besides, the proposed metrics can be used for identifying relevant events for authors by providing a flexible filtering mechanism that comprises various selection criteria. This way, authors can be offered plausible recommendations for their intended publications that match quality demands, community guidelines, or other personal interests best. For example, authors can determine the best fitting conference for increasing their h-index (FM12.2) or find suitable alternatives for a rejected paper.

*Organizers* Organizers of scientific events can benefit from the proposed metrics as they allow for monitoring the quality and impact of their events to the research communities over time. It is even possible to estimate fundamental metric values that allow for improving the reputation (or rank) of an event in a systematic way. For example, the reputation of publishers (DM6.3), such as Springer and ACM, may change over time, and event organizers can adapt to this development. The same applies to paper indexing services, such as ISI/SCI and DBLP, because a good choice can enhance the popularity of events. In addition, the introduced metrics support organizers in determining new hot research topics that should be covered in future events and help with finding suitable PC members, chairs, or keynote speakers. For example, the information about a continuous increase of submissions related to a certain topic indicates a growing interest in the respective research community, which can be used to adjust the focus of a conference.

*Community* Aside from assisting authors and event organizers in evaluating the current status, most of the proposed measurements are temporal values, and the corresponding history allows for a comprehensive retrospective analysis of their developments. For example, an event can benefit from the fact that the values of positive indicators increase over time, while negative ones should decrease. In addition, the stability of certain positive aspects such as the number of submissions and participants, size of the PC, or changes to the steering committee becomes transparent and can be used for quality evaluation. Having a history of our proposed metrics at hand allows for projecting the likely development of certain values in the future. For example, a continuous increase in the impact factor FM12.1 of a scientific event may attract the interest of authors because it might indicate the development of a new top reference. Figure 4 shows the relevance of the defined metrics to different stakeholders of scholarly communication. The audience is categorized in roles in the whole workflow of scholarly communication, such as reviewers and organizers.

## Implementation

**Fig. 3:**
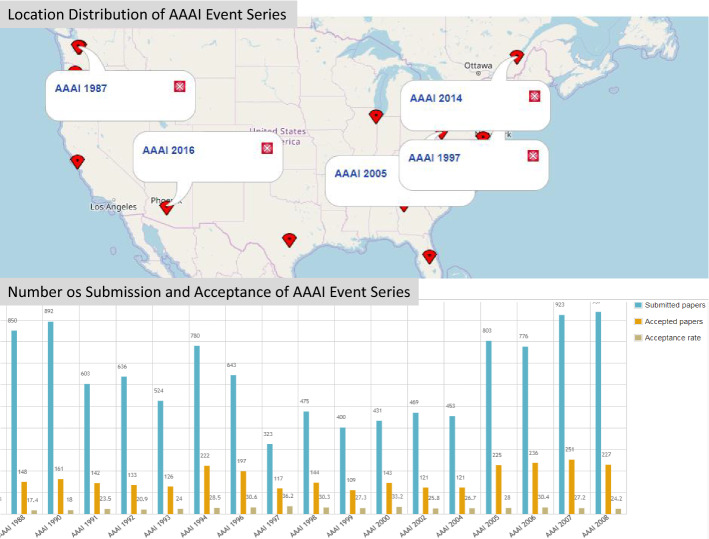
Distribution of event series AAAI over time and variation of acceptance rate considering the number of submissions and acceptances

OpenResearch.org^21^ (OR) is a crowdsourcing platform that we developed to assist researchers in collecting, organizing, sharing, and disseminating information about scientific events (Vahdati et al. 201). It enables quality-related queries over a multidisciplinary collection of events according to a broad range of criteria such as acceptance rate, the sustainability of event series, and the reputation of people and organizations. The purpose of this work is to provide a comprehensive and potential list of metrics to be implemented in OR. At the time we conducted our research (2017), some of the metrics such as calculation of acceptance rate, distribution over time, distribution by location, categories of events by topics have been implemented. For most of the high-level metrics, we will have to use SPARQL because of the limitations of the MediaWiki expressions, for example, person reputation and roles. Figure 3 shows an example^22^ of visualization of a composite indicator, i.e., a derived metric, defined in the template and bound with its corresponding extension.

Implementation of the defined metrics and dimensions has been done with an on-demand decision-making process. Some of the metrics suited to be defined as a raw property. The derived metrics have been computed by queries over the data (using MediaWiki expressions), for example, $acceptance rate := accepted / submitted$; average acceptance rate over series). The implementation of this composite indicator that can be calculated from the raw properties has been done in the template of the corresponding entity(here event): 
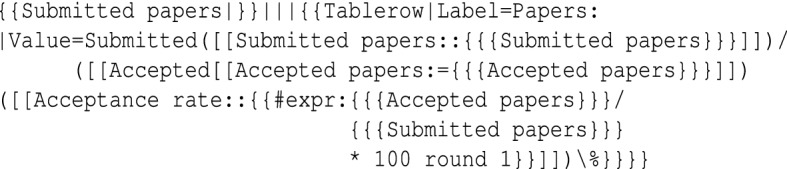


The type of implemented metrics is specified by adding the type acronym in front of each metric shown in Table 1. Composite indicators that are added in the template are labeled by *temp*, raw properties as *prop*, and some of the metrics have been added as categories (*cate*). For most of the high-level metrics, we had to use the inline queries^23^ by the expression language of SMW, these types of implemented metrics have been specified as *ASK*. For some of the metrics, because of the limitations of the MediaWiki expressions, for example, person reputation and roles, implementation was only possible using the more expressive SPARQL query language. These types of metrics have been labeled as *SPARQL*. Those metrics without labels are yet to be implemented. The *ASK* query given below computes currently ongoing events.

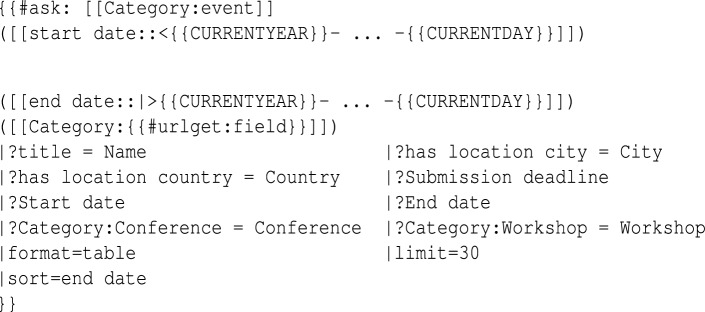



## Evaluation

This section describes three methods that were used to show the value of the proposed quality assessment framework and its implementation in the OpenResearch.org platform. To evaluate the *necessity* of having such a systematic way of assisting scholarly events, we conducted two surveys among a group of computer science and social science researchers. For the first survey, we invited researchers with different roles in scientific events with the aim of assessing the importance of the need for a systematic management of scholarly metadata about events. We designed the questions with the objective of finding out whether researchers agree on the necessity of having such services. The survey addressed two main aspects: 1. what criteria are relevant to find suitable events, and 2. how can we evaluate an event manually. The second survey addressed the *usability* of the OR platform for managing structured information about scientific events.

We also conducted an evaluation by applying the quality metrics defined in section “Quality metrics” to a selection of top computer science events in order to evaluate whether the metrics correlate with an intuitive perception of an event as “high quality”. In the fourth evaluation, we showed how the developed platform could assist researchers by providing valuable answers to queries using a combination of the defined quality metrics.

### Necessity survey

We asked 60 researchers from two scientific fields, computer science and social science, to explain 1. the most important metrics for their community to find and select a scientific event, and 2. the current ways they use to explore scientific events based on these metrics. The findings of this study are explained below (the data is available online^24^).

*Finding relevant and good events* Participants indicated that they explore scientific events using search engines, mailing lists, social media, and personal contacts. Then, they assess the CfPs to find out whether an event satisfies their criteria. For selecting an event to participate in, all participants confirmed that they consider information that is not served directly by the current communication channels.

*Agreement with our metrics* More than half of the participants agree that (from the criteria defined in section “Quality metrics”) the main criteria that make an event the best event of its scientific community are: the quality of reviews (M3, M4), the reputation of organizers and keynote speakers, the topical focus of the event, and a high number of citations of the papers accepted in previous years. Additionally, they confirmed the relevance of the following criteria: location (M2), networking possibilities (M13), review quality (M3, M4), the reputation of the organizers (M16), keynote speakers and sponsors (M16), acceptance rate (M4), the quality of co-located events (M16), the accessibility of the location (M16), and citations counts for accepted papers of previous years (M16), i.e., the “impact factor” (M16).

*Most valuable metrics* Overall, 95% of the participants agreed that the quality metrics defined by us can lead to a quality-based ranking of scientific events. For some metrics, it is not straightforward to *interpret* whether the raw, measurable value is good or bad. Concretely, we asked the participants to characterize the metrics in the “review” dimension accordingly. 90% of the participants of the best conferences in their area of research received 3 or 4 reviews (including meta-reviews) per submission. Over 77% of the participants estimated the average length of the reviews between 10 and 30 lines of text.

*Relevance of a recommendation service* Over 36% of the participants agreed that having an event recommendation service is highly relevant for them. 46% of them answered it is somewhat relevant.

**Fig. 4:**
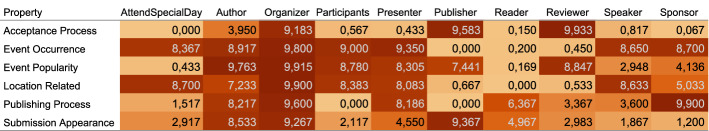
The relevance of the defined metrics to different stakeholders of the scholarly communication

*Relevance of a defined metrics to person roles* We grouped our metrics into six high-level categories including different dimensions: submission appearance, acceptance process, location, event occurrence, event popularity, and publishing process. Survey participants were asked to score (0 being the lowest and 10 the highest rank) the relevance of these categories to groups of stakeholders. Results are shown in Fig. 4.^25^

It is straightforward to see that all categories are relevant to event organizers, whereas the focus of readers is most selective.

### Usability survey

The main goal of the second survey was to determine to what extent the OR platform is usable for the community, including its CRUD functions (create, read, update, delete Martin 1983) supporting the tasks of participants using the platform. Participants were asked to evaluate OR’s usability based on their experience. Twenty out of 60 participants of the first survey volunteered to use OR and participate in the second survey.

*Background of the participants* Participants of the second survey have had several roles in scientific events (participant, PC member, event organizer, and keynote speaker). Before working with OR, 18 of them had had basic knowledge about wikis but not about Semantic MediaWiki.

*Ease of Use* 75% of the users replied they had basic knowledge about wikis in general; however, half of them did not know about Semantic MediaWiki, the software driving OR. 66% got familiarized easily with OR, which shows its suitability for researchers of different fields. The same number of participants answered that they needed less than 5 min to add a single event, which is a relatively low time regarding the time organizers need to announce their event in several channels. The average number of single events created by individual users is 10. More than half of the participants needed less than 5 min for a bulk import. The participants largely agreed that these times are reasonable.

*Ease of event publicity using OR* We also asked the participants to answer how long it takes to announce an event by circulating emails over mailing lists and social media posts. The participants with organizer roles answered they either spend few days (1–2 days) or some hours, including formulating the announcement email and sending it through different mailing lists as well as keeping the social media posts up to date.

*OR’s performance* In the second phase of the online experiment, those 20 out of 60 survey participants who were familiar with OR were asked questions about its usability and effectiveness. The average score they assigned to the ease of importing data into OR using Semantic Forms and bulk imports was 3.5 in the range of 1 (not easy at all) to 5 (very easy). Seventeen participants reported that the data import took them between 5 and 10 min.

*Intention to future use of OR* More than half of the participants declared their intent to import events into OR whenever there was a related event that had not yet been added to OR. Almost half of the participants said they would try to add relevant events at least every 2 weeks.

### Event metadata evaluation by applying quality metrics

We evaluate our quality metrics for scientific events by applying them to events in our own community, i.e., events of whose quality we have an intuitive understanding, and then discuss our observations. Table 2 lists the conference series we considered in providing the statistics. These are either top-ranked conferences or often targeted venues from the majority of the community who participated in our surveys. For evaluating the application of metrics to event series as well, we studied the past ten editions of the for WWW (TheWebConf) and VLDB conference series because of data availability. In the following, we summarize the results of the assessment per dimension.^26^

**Table 2:** List of the observed conferences in 2015 (sorted by rank)

Acronym	URL	Community ranking	Suggested by**	Publisher
WWW	https://www.iw3c2.org	A*	19	ACM
SIGMOD/PODS	http://sigmod2015.org/	A*	8	ACM
VLDB	https://www.vldb.org	A*	6	VLDB
JCDL	http://jcdl.org/	A*	2	ACM
ISWC	http://swsa.semanticweb.org/	A	18	Springer
ESWC	http://eswc-conferences.org/	A	14	Springer
WI	http://wi-consortium.org/	B	2	IEEE
SEMANTiCS	http://semantics.cc/	–	6	Springer
KESW	http://kesw.ru/	–	2	Springer
WIMS	http://wims.vestforsk.no/	–	2	ACM
ICSC	http://ieee-icsc.org/	–	2	IEEE

**Suggested by the participants of the first survey from computer science community

*Submissions* All observed conferences accepted submissions in the LaTeX and MS Word formats. Two conferences, ESWC and ISWC, have started to accept submissions after 2018 as Web-centric articles in HTML format providing better accessibility and potential for interactivity. All of the conferences followed their publisher’s document style. Four conferences accepted submissions in ACM style, four in LNCS style, and the remaining two in IEEE style. VLDB has its own style. Overall, flexibility concerning style is limited by the publishers, and LaTeX and MS Word still dominate the accepted formats. This proves that scientists still write static documents, which do not use the possibilities of digitization, such as interactivity, easy accessibility, multimodality, or semantic content annotation and representation (Capadisli et al. 2015; Peroni et al. 2017). Besides the positive examples of ISWC and ESWC accepting HTML since 2018, such innovations have not been pioneered by other big conferences so far. The SAVE-SD workshop co-located with WWW 2015 allowed LaTeX and MS Word but preferred submissions in RASH (Peroni et al. 2017). As data about authors’ affiliation is rarely available in an open, structured, reusable form but often hidden in the databases of submission management systems such as EasyChair,^27^ it is hard to determine the diversity of submissions with regard to countries. Determining diversity with regard to sectors is harder: while an author’s country is usually recorded explicitly, one would have to infer the sector from the author’s organization.

*Location* Table 3 shows the number of times event series considered in this study visited different continents. ESWC is a European conference obviously never held out of Europe. The same fact is true for KESW as an east European conference has been held in Russia seven times out of overall eight editions and once in Poland. ISWC, which had its 17th edition in 2018, has been held five times in Europe, six times in the US, four times in Asia and three times in Australia. VLDB has visited a wide range of visited locations, including many countries per continent, while WWW (TheWebConf) is selective about countries. Neither of them has visited the same continent for two successive editions.

**Table 3:** Distribution of conferences over continents

Conference	Overall	America	Europe	Asia	Australia
ESWC	16	0	16	0	0
ICSC	13	12	1	0	0
ISWC	18	6	5	4	3
JCDL	17	15	1	0	1
KESW	6	0	6	0	0
PODS	36	31	3	1	1
SEMANTiCS	15	0	15	0	0
VLDB	43	12	20	9	2
WI	18	6	6	5	1
WIMS	9	0	8	1	0
WWW	26	10	10	5	1

To reduce the number of zeros, North America and South America are presented as one and Africa is skipped

**Fig. 5:**
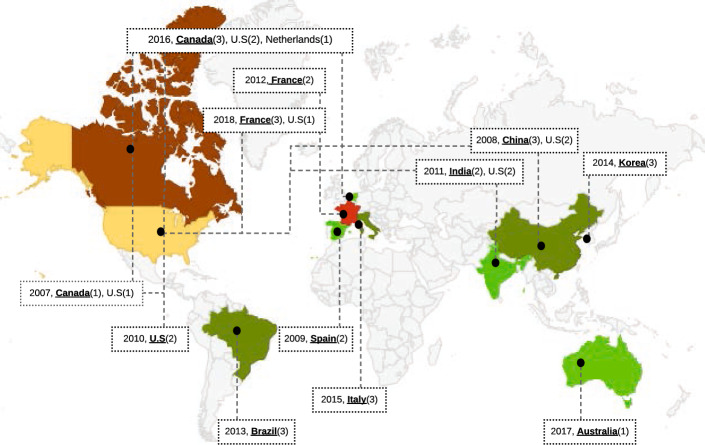
Relation of event location and people organizing the event, looking at last 11 years of the WWW conference (TheWebConf)

*Review process* The majority of computer science conference proceedings are peer-reviewed, in particular those in our evaluation. A few conferences, particularly WWW (TheWeb) and SIGMOD/PODS, perform double-blind reviews. ESWC 2018 has introduced a fully open review policy with making submitted papers publicly posted on the ESWC web site. In a fully open review process, the authors are not anonymous to the reviewers, while the reviewers can be known to authors by default.

**Fig. 6:**
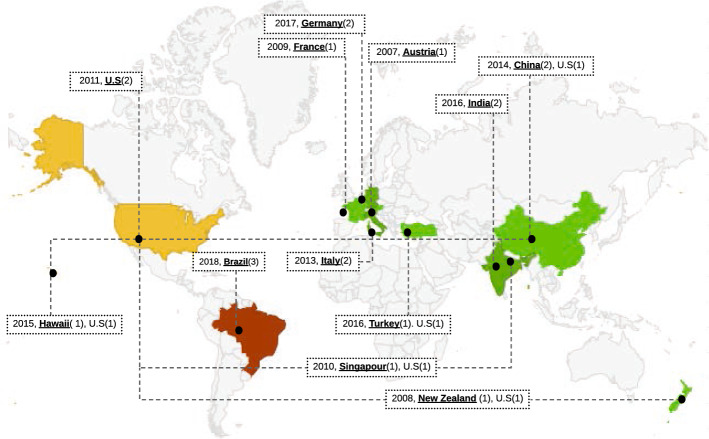
Relation of event location and people organizing the event, looking at the last 11 years of the VLDB conference

**Fig. 7:**
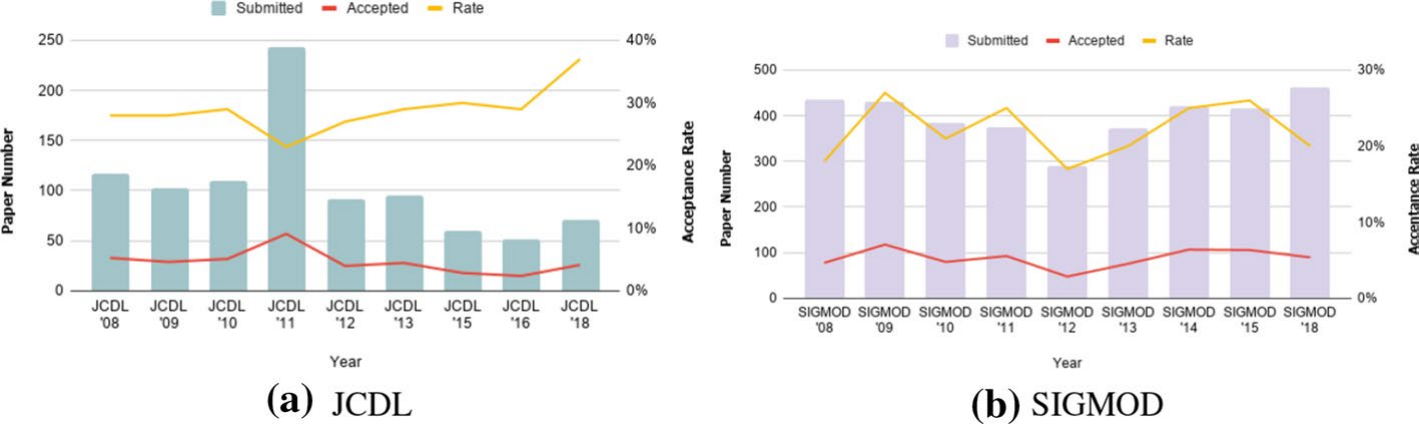
JCDL and SIGMOD/PODS paper acceptance rate and number of submitted/accepted papers for the last 10 years

*Review results* The acceptance rate of WWW (TheWeb) 2018 was 17%; in the previous editions, it has always ranged between 11% and 19%. VLDB maintains a dedicated statistics page.^28^ The acceptance rate for the research track has always been below 20%. The number of papers submitted to JCDL has dropped significantly in the last ten years. The submission number was 117 in 2008 and decreased to 71 in 2018; however, the acceptance rate had increased from 28% in 2008 to 37% in 2018. The number of papers submitted to SIGMOD/PODS was between 250 and 500 in the last ten years. Acceptance rate fluctuated between the values 18–27%; however, in 2018, this conference accepted 20% of the papers submitted. Figure 7 shows the last 10 years of change in the accepted number of papers for the JCDL and SIGMOD/PODS conferences. For the other conferences, the acceptance rate varied between 22 and 32%. Information about accepted and submitted papers can be found from the proceedings of conferences in the ACM Digital Library.

Further information about the review result is not public for the assessed conferences. We therefore asked ten researchers in the community who had submitted to any of these conferences to provide us with information on the reviews they had received regardless of acceptance or rejection. Based on this data, ESWC 2017 had four reviews plus one meta-review per submission. All the other conferences provided at least three reviews per submission, of which around two were of sufficient quality. The average length of WWW 2017 and VLDB 2017 reviews was more than 100 lines of text, which indicates high quality. Regardless of whether papers were accepted or rejected, the average review length in four more conferences was more than 50 lines per review, and the authors considered them helpful, which emphasizes the expertise of the reviewers. For the remaining four conferences (ICSC, WI, WIMS, and SEMANTiCS), the average length of the reviews were below 25 lines. Surprisingly, reviews from the KESW conference have been ranked highly with regard to the length and quality of the reviews, and their was an average of three reviews per submission. On the other hand, its acceptance rate has usually been over 35%, but surprisingly dropped to 28% in 2017.

*Publishing* The reputation of the publisher and the expected impact of its publications have a great influence on the decision of researchers to submit to an event or to read its proceedings. All of the conferences publish with one of the major commercial computer science publishers: ACM, IEEE, Springer. Not all of the co-located events used the same publisher as the main event. In some cases other publishers such as Elsevier^29^ or IOS Press^30^ were chosen. The VLDB conference series uses its own publishing process without having an external publishing house involved. ACM is the major publisher for the conferences evaluated in this study; however, electronic versions are included in both the ACM and IEEE digital libraries.

*Discoverability* The results we obtained when searching by topic prove that without being aware of the existence of a particular event, one will hardly discover it. We did find related *journals* while searching by topic (e.g., the Semantic Web Journal when searching for “semantic web”) among the top 10 results, but none of our evaluated conferences. The ranking of every conference improved significantly by adding the type of the event, i.e. “conference”, as a search keyword. Specific keywords related to the main topic of the event, such as “database management” and “database research” for SIGMOD or “digital library” for JCDL with using the “conference” keyword, are helpful to find the exact conference. However, conferences related to web intelligence were not included in the search results. In addition, the homepage of every event evaluated made it into the top 10 results by adding the year (here: “2018”) to the acronym of the event.

**Fig. 8:**
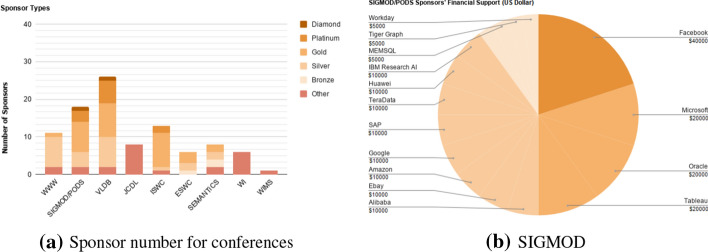
Distribution of sponsors’ contributions, and amount of financial support of SIGMOD/PODS in 2018

*Sponsorship* The big players of the community, including Google, Facebook, and Microsoft, typically sponsor big events. Figure 8 shows the distribution of sponsor’s contributions to SIGMOD 2018. Additionally, the bar chart illustrates the number and distribution of sponsors for each conference in 2018. Same colors are used for the same level of sponsorship in both charts; sponsors are categorized in diamond, platinum, gold, silver, bronze, and other. VLDB had the highest number of sponsors (more than 30) in 2018 when compared to other conferences in the bar chart. VLDB, SIGMOD/PODS, and SEMANTICS conferences had four levels of sponsors; JCDL, WI, and WIMS used different levels. The areas of the SIGMOD pie chart reflects the overall amount of financial support. The largest area shows the platinum sponsorships, which has the highest amount of support ($40,000 or over). Smaller areas show the gold ($20,000), silver ($10,000), and bronze ($5000) sponsors of the SIGMOD 2018 conference, respectively.

WWW (TheWebConf) 2018 had two local sponsors, i.e., Inria and Idex Lyon, which aimed at increasing their popularity in academia by taking advantage of the proximity of the event’s location. Our data shows a strong correlation between sponsors and organizers; in most cases, at least one organizer has a role in an organization or a project that sponsors the event.

*Reputation* According to Google Scholar, the h5-indexes of WWW (TheWebConf), VLDB, SIGMOD, ISWC are 70, 74, 67, and 37, respectively.^31^ No such information is available for the other conferences. According to the CORE2018 ranking, WWW, VLDB, PODS, and JCDL are A* conferences, while ISWC and ESWC are A conferences, and WI is a B conference; the remaining ones are not ranked.^32^ However, the community considers them as important events that it is worth to submit their work to.

*Co-location* The co-located meetings are seen as a gathering of innovators. They offer participants opportunities to discuss ongoing work and late-breaking results, as well as shaping new ideas and research fields. All the conferences organize affiliated workshops, symposiums, and tutorials as co-located events; some of the big conferences even provide co-located conferences. WWW 2018 (TheWebConf) offered co-located conferences inside its main program: International Conference on Digital Health, BIG 2018, and the Web4All conference. BIG 2018 was organized as a track for the participants, i.e., it followed the same registration process and submission rules as the WWW 2018 conference. In general, however, such conferences can be location-specific and can have their own website and different registration process from the main conference.

*Registration* The registration process is generally managed by online registration forms. For all conferences listed in Table 2, people redirected to the registration website to fill the forms. Registration can be made early or late according to the date. Early registration is cheaper than late and regular registration. Some of the conferences also accept onsite registration. The registration fees of the VLDB conference in 2018 were $700 for early, $800 for regular, and $900 for onsite registration, and they offered discounts to students. Besides, JCDL gave discounts to members of certain societies in 2018. Moreover, ISWC, ESWC, and WWW (TheWebConf) gave discounts just for students. Registration fees can change with respect to attendance. People could register for one day of the conference; for example, JCDL offered one-day attendance at a lower price in 2018. Daily passes were an option at WWW (TheWebConf) 2018 if someone wanted to attend the conference for specific dates.

**Fig. 9:**
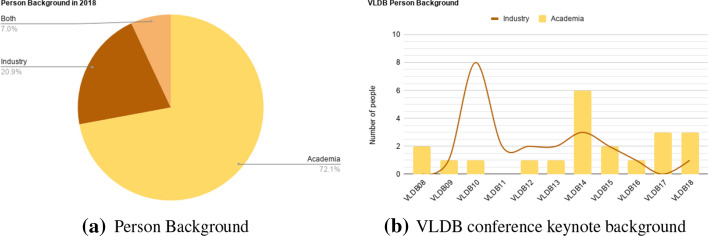
Keynote speakers’ background percentage distribution in 2018 for all conferences in Table 2, and VLDB conference keynote speakers background distribution for the last 10 years

*Schedule* The program of the conference gives the details of the events that take place at a specific time. The program schedule is usually displayed on conference’s website or downloadable as a PDF document. The time, place, topic, and speaker of the event can be seen from the conference schedule. The program schedule involves detailed information about workshops, tutorials, panels, keynote speakers, award sessions, and breaks. Big events can usually accommodate 2–4 workshops or tutorials daily, and these events can run in parallel. Events of JCDL and VLDB in 2018 started at 8:30 in the morning and ended at 18:30 or 20:30. In contrast, ISWC and ESWC conference sessions generally started at 9:00 or 9:30 and ended at 16:00 or 17:00. Most conferences had parallel sessions. Most of the conferences include a lunch break and a 30-min coffee break twice a day.

*Participants* Every WWW conference from 2006 to 2018 has recruited at least half of their general chairs from the country where the conference was located. WWW generally has PC members with a high reputation, who demonstrate further commitment in that they often organize co-located sub-events. The h-index of people involved in the WWW series ranges from 15 to 90; their i10-index ranges from 20 to 500 with up to $\sim$30,000 citations. The frequency of involvement of PC members and keynote speakers is high, but the organizers vary. For example, Tim Berners-Lee, the founder of the area, has been the keynote speaker of six editions of the WWW conference. All academic keynote speakers of the WWW conferences evaluated had an h-index of over $\sim$25 with more than $\sim$1000 citations. Most of the above facts similarly hold for VLDB. Industrial keynote speakers of WWW and VLDB are founders of big players, heads of big companies, etc. Every edition of WWW and VLDB over the past 10 years had around 900 registered participants. The other eight conferences evaluated pursue a different strategy. They have a core team of people frequently involved in the organization, whereas the frequency of involvement of PC members and keynote speakers in the same role varies.

*Person role* The most prominent finding from the analysis is that the organization committee members, as well as keynote speakers, are changing when the event location changes. However, the core part of the program committee largely remains the same, with slight changes from local scientists being introduced. Distribution of general chairs with regard to the location of the two conferences (WWW and VLDB) from 2007 to 2018 is shown in Figs. 5 and 6. In both of these event series, people happen to take several roles in the same event edition editions (e.g., one person was an organizer and an author in WWW 2018) or different roles in different editions (e.g., one person was an organizer in WWW 2017 and an author in WWW 2018). In more than 20 cases out of 40, keynote speakers have also had the role of program committee members. Looking at the list of keynote speakers of the above conferences during the last ten years, at WWW, 30% of them were from academia, 60% were from industry, and 10% were from both academia and industry (according to the affiliation given on the conference homepage or in their Google Scholar profiles). However, at VLDB, 48% of the keynote speakers have been from academia and 52% from industry, as shown in Fig. 9. The SEMANTiCS event series has more than 60% of its keynote speakers from industry; the opposite holds for JCDL. Figure 9 also shows the percentage distribution of keynote speakers in 2018 of all conferences in Table 2 considering academia, industry, and both academia and industry backgrounds. People from academia make up the highest percentage of approximately 72%, while there are approximately 21% from industry. People from both academia and industry have the lowest percentage of 7.0%.

**Fig. 10:**
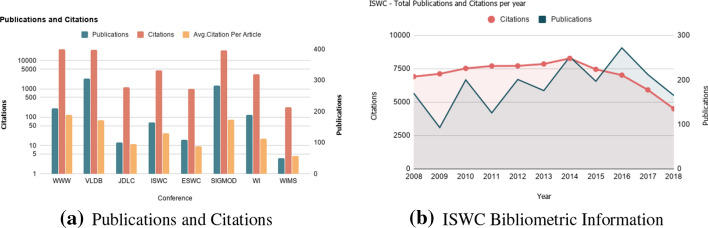
Publications-citations numbers and bibliometric information of ISWC for the last 10 years

*Bibliometrics* Bibliometric analysis of each conference for the year 2018 is depicted in Fig. 10; it varied according to the total number of published articles. The total number of publications and citations, covering ten years from 2008 to 2018 for each conference, was exported from Microsoft Academic.^33^ Based on the bibliometric analysis of each conference, VLDB and SIGMOD have the highest number of publications, 306 and 283, respectively. Based on the total citations count, WWW, SIGMOD, and VLDB are high impact events in comparison to others. These three major conferences also contain most-cited articles as measured by the metric of the average number of citations for an individual article. In addition, we examined the ISWC conference based on bibliometrics data, stated in Fig. 10, for the last 10 years. It appears that the total number of citations does not increase proportionally with the number of publications. For example, the number of published articles raised from 197 to 272 between 2015 and 2016, while the number of total citations dropped from 7459 to 7027.

*Publicity and social impact* Conferences make announcements on social network platforms, such as Twitter, and Facebook to reach participants. Seventy percent of the conferences have Twitter and Facebook accounts; however, only the SEMANTiCS’18 conference used additional platforms such as LinkedIn, Google Plus, and Xing. They also put meaningful hashtags in their Twitter accounts to increase the visibility in their tweets and to classify them in a way that makes it easy for other users to find and follow tweets about a specific conference. Most of the hashtags include the conference acronym and the year of the conference such as “#eswc2018, #jcdl2018”; on the other hand, some conferences use just the name of the conference, e.g., “#iswc_conf, #semanticsconf”. Regarding website quality, content and style typically follow a conference theme. All 11 conferences have a website and provide a different web page for each year. They listed all past conferences with links on an archive or history tab to track data of past events. Especially the websites of WWW 2018 and SIGMOD 2018 conferences provide a visually appealing design to users and contain clear navigation through the tabs. The other important point is that most websites use the same terminology in the navigation menu, such as “Call for papers”, “Submission of Papers”, etc., which makes it fast to find relevant information. The main considerations of content regarding readability and other usability aspects contribute to attracting more participants and have a crucial effect on the users.

## Conclusion and future work

We proposed a framework of assessment metrics that allows for a differentiated quantification of the quality of scientific events and publications. This supports authors in finding the most suitable event for their publications according to various motivation criteria. In particular, young or even unknown events can benefit from that and can become attractive for authors because of certain quality metrics despite being still poorly ranked in general.

Organizers can also benefit from our framework because the quantification of quality becomes transparent and possibilities to positively influence the quality metrics can be systematically explored. In addition, the temporal evolution of the assessment metrics can be investigated, which allows for identifying the stability of certain quality measurements such as acceptance rate. This plays an essential role for sponsors and long-term partnerships.

Since there is no centralized and comprehensive data source, we still need to accumulate event-related information from different sources (such as online statistics, homepages, DBLP and Google Scholar, ranking pages, Wikipedia, etc.).

One part of future work is the development of software that helps to automate this data accumulation procedure and the subsequent curation. This is the main focus of our ConfIDent research project^34^ (2019–2022). As most of the initial ConfIDent metadata schema was developed during the COVID-19 pandemic, when some conferences were canceled but most actually changed from physical to virtual events,^35^ we have started collecting information on the possibility of remote participation. Virtual events have a significantly lower cost than physical events, and thus typically lower registration fees and less of a need for sponsoring. Thus, we are observing the emergence of new quality metrics based on new properties of conferences, as well as a shift of importance of some of the quality metrics we have defined in our work. Subsequent tasks in ConfIDent aim at investigating these developments.

In our evaluation, we asked the participants to characterize the metrics in the “review” dimension according to what values are good or bad; doing so is actually necessary for a lot more metrics (such as acceptance rate). In this way, we can differentiate major dimensions from minor ones in order to determine quality measurements with certain accuracy.

We furthermore plan to improve the OpenResearch ontology by a formal model of our event quality metrics, adapting the structure of our previously developed ontology for *data* quality metrics (Debattista et al. 2014). We will do evaluations on fake conferences to confirm our metrics help distinguish fake from serious conferences.

## Footnotes

1 https://5stardata.info/en/.

2 https://www.springernature.com/gp/researchers/scigraph.

3 http://scholarlydata.org/dumps/.

4 http://ceur-ws.org/HOWTOSUBMIT.html.

5 https://github.com/ceurws/lod/wiki.

6 http://iso.org/iso/news.htm?refid=Ref1598.

7 http://www.core.edu.au/conference-portal.

8 https://scholar.google.com/intl/en/scholar/metrics.html.

9 http://wikicfp.com/cfp/.

10 http://paperleap.com/.

11 https://research.cs.wisc.edu/dbworld/.

12 “An aggregate function is a function where [multiple values] are grouped as input on certain criteria to form a single value of more significant meaning” (https://en.wikipedia.org/wiki/Aggregate_function).

13 http://jianying.space/conference-ranking.html.

14 https://chrome.google.com/webstore/detail/live-shine-beta/obohlnnfogkjeknnfgcfmacjbgekmana.

15 https://scholar.google.com/citations?view_op=top_venues&hl=en.

16 http://www.simpleweb.org/wiki/Conference_Ranking.

17 https://dl.acm.org/ccs.

18 https://zenodo.org/.

19 https://arxiv.org/.

20 http://f1000.com/prime.

21 http://openresearch.org/wiki/.

22 http://openresearch.org/wiki/AAAI.

23 https://www.semantic-mediawiki.org/wiki/Help:Inline_queries.

24 https://goo.gl/DSSazs.

25 Drawn by Tableau: https://www.tableau.com.

26 The full raw data is available at https://bit.ly/2ApVtTR.

27 http://www.easychair.org/.

28 http://www.vldb.org/archives/statistics.html.

29 www.elsevier.com.

30 https://www.iospress.nl/.

31 https://scholar.google.de/citations?view_op=top_venues&hl=en&vq=eng_databasesinformationsystems.

32 http://portal.core.edu.au/conf-ranks/.

33 https://academic.microsoft.com/home.

34 https://projects.tib.eu/en/confident/.

35 https://www.acm.org/virtual-conferences.
